# The Incidental Dermoid: A Rare Cause of Small Bowel Obstruction in a Patient With Simultaneous Renal Cell Carcinoma

**DOI:** 10.7759/cureus.101939

**Published:** 2026-01-20

**Authors:** Sneha Choudhary, Parul Talwar, Sabah Fatima, Shashank Mishra, Manoj Andley

**Affiliations:** 1 Surgery, Lady Hardinge Medical College, New Delhi, IND; 2 Obstetrics and Gynecology, Bhagwan Mahavir Hospital, Delhi, IND

**Keywords:** dermoid cysts, incidental finding, renal cell carcinoma, small bowel obstruction, synchronous tumors

## Abstract

Benign ovarian dermoids are a common incidental finding in adult women. Presentation with small bowel obstruction (SBO) is rare and creates diagnostic uncertainty. Here, we describe the case of a 69-year-old lady with small bowel obstruction, who was incidentally diagnosed with a right adnexal mass and right renal mass on imaging. The correlation between bowel obstruction and the adnexal mass was unknown until laparotomy, which showed dense adhesions between the ileal loops and the adnexal mass. Following the creation of a temporary loop ileostomy, definitive management was done four months later and included adhesiolysis, right oophorectomy, and right nephrectomy. Postoperative histology revealed an inflamed and ulcerated mature cystic teratoma of the ovary, which was the cause of the patient’s symptoms, and a renal cell carcinoma of the right kidney. With the universal application of cross-sectional imaging, the knowledge of such unusual associations can help surgeons decipher the occasional clinical relevance of incidentalomas. Additionally, to the best of our knowledge, this is the first documentation of a renal cell carcinoma synchronous with a benign ovarian dermoid cyst.

## Introduction

Small bowel obstruction (SBO) represents a sizable component of the clinical profile of patients requiring urgent surgical intervention. Globally, the most common cause cited is postoperative adhesions [1]. In patients without a history of prior surgery, the more frequent etiologies include obstructed hernias and malignancy, with abdominal tuberculosis being an important cause in India [1].

Ovarian dermoid cysts constitute a rare cause of SBO, whether due to adhesions, rupture, fistulization, or mass effect, with only a handful of such cases reported previously in the literature [2-6]. In adult women, however, these are known to be common incidental findings, adding to the diagnostic difficulty in determining the association between such seemingly unrelated surgical pathologies.

## Case presentation

A 69-year-old postmenopausal lady without a history of previous surgery or known comorbidities presented to the emergency service with progressive symptoms of abdominal colic, obstipation, bilious vomiting, and decreased urine output over five days. She was afebrile with a heart rate of 120 beats per minute (bpm), blood pressure of 84/50 mmHg, and SpO_2_ of 98%. Examination revealed a soft, distended abdomen with guarding and absent bowel sounds. A complete blood count was within normal limits, and renal function tests showed the presence of hyponatremia and raised serum creatinine, reflecting fluid loss.

**Figure 1:**
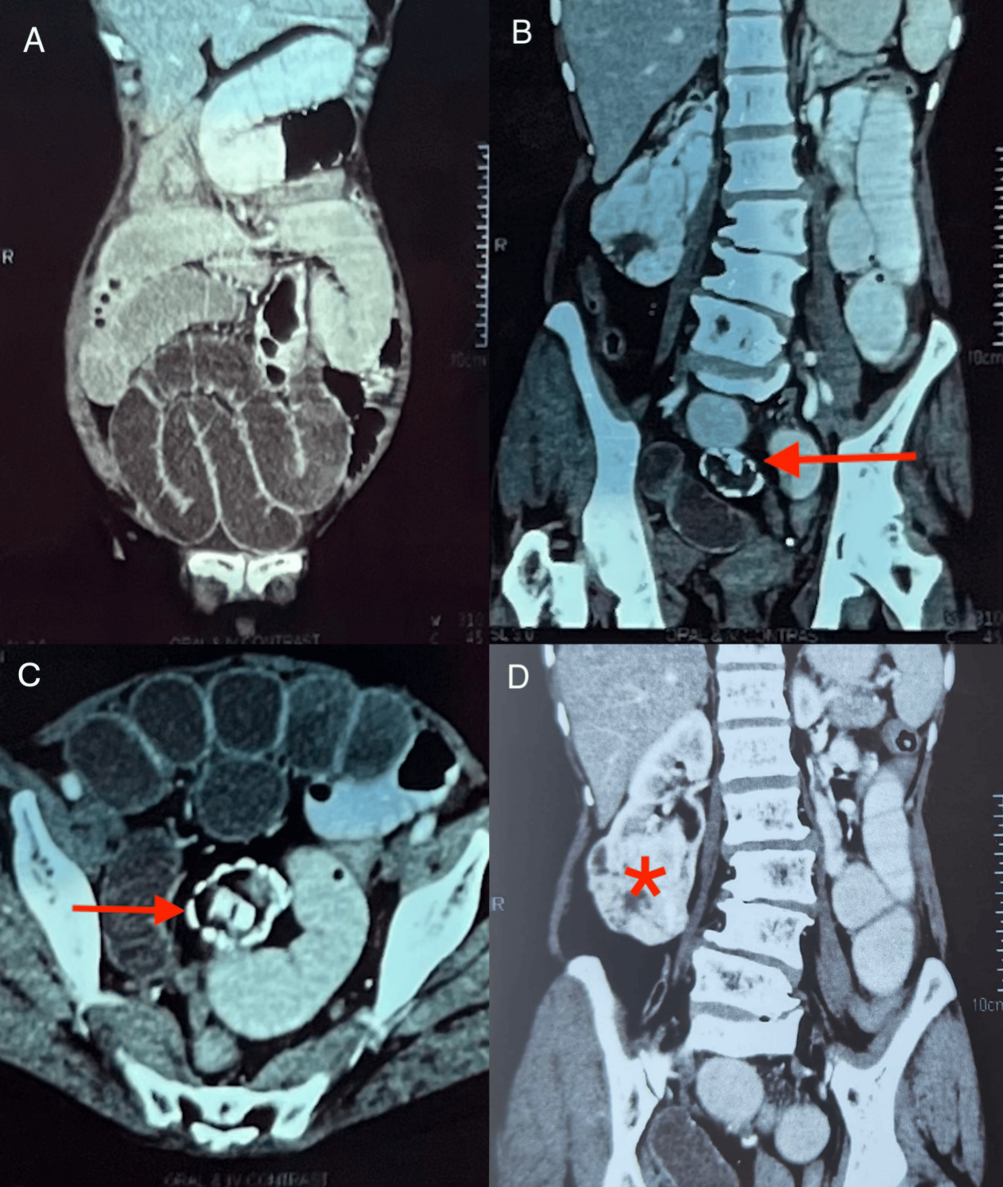
Abdominal CECT with oral and intravenous contrast (A) Coronal image showing dilated small bowel loops. (B and C) Coronal and axial views showing a heterogeneous mass (red arrow) with fat attenuation and peripheral calcification in the lower abdominal region abutting the dilated small bowel loops, suggestive of a superiorly displaced right adnexal mass. Mild sclerotic and degenerative changes in the lumbar vertebrae are present. (D) Coronal view showing a well-defined, heterogeneously enhancing exophytic solid mass lesion (asterisk) with necrosis in the lower pole of the right kidney CECT: contrast-enhanced computed tomography

An abdominal contrast-enhanced computed tomography (CECT) (Figure 1) gave the impression of mechanical SBO with dilated small bowel loops (Figure 1A) and mild free fluid in the lower abdomen and pelvis. Additional findings included the presence of a 4.0 × 3.6 cm heterogeneous right adnexal mass abutting the small bowel loops (Figure 1B, 1C) and a 5.8 × 5.4 × 6 cm well-defined, heterogeneously enhancing exophytic mass with necrosis at the lower pole of the right kidney (Figure 1D).

Fluid resuscitation was initiated, and an emergency laparotomy was performed in view of SBO, which had not responded to conservative management. Intraoperative findings included extensive adhesions surrounding the distal ileum and the adnexal mass, along with the confirmation of the renal mass. Limited adhesiolysis and peritoneal lavage were carried out, and a decompressive loop ileostomy was created with a plan to restore bowel continuity and remove the incidental masses at a later date.

A gynecology opinion was obtained, and based on imaging features and normal cancer antigen 125 (CA-125) levels, the adnexal mass was characterized as benign. Four months later, after nutritional optimization, a normal distal loopogram, and a preoperative workup negative for tuberculosis, the patient was taken up for right nephrectomy, right oophorectomy, and ileostomy closure.

Radical right nephrectomy was performed first and without incident, following which attention was turned to the ovarian mass. Small bowel loops were seen densely adherent to each other and to the cystic adnexal mass. The adhesions were released, and a right-sided oophorectomy was performed. The closure of the ileostomy was performed with a side-to-side stapled ileo-ileal anastomosis. The postoperative course was uneventful, and the patient is doing well after a follow-up of six months.

**Figure 2:**
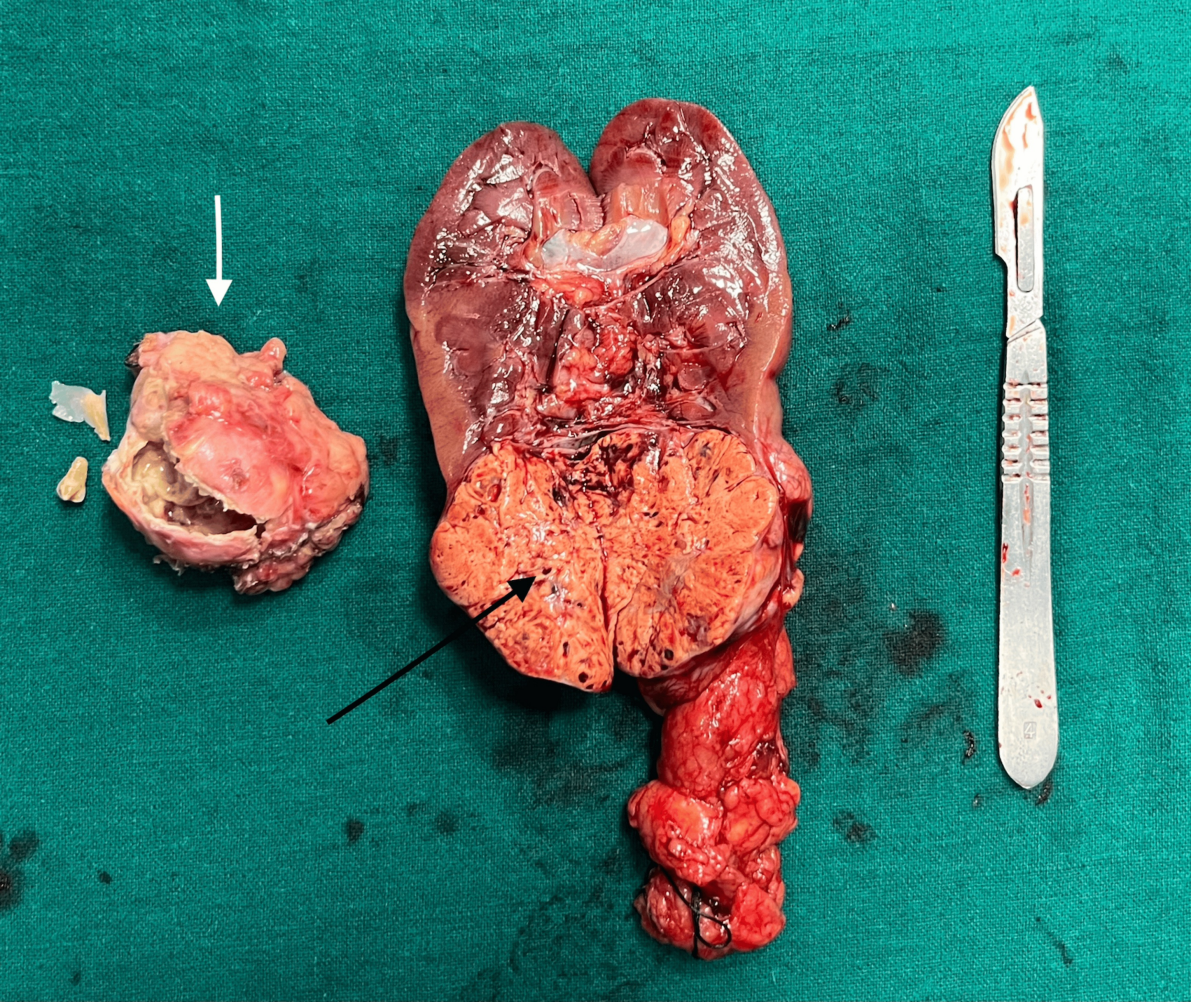
Specimen examination showing adnexal cyst (white arrow) with irregular wall; contents included a tooth, bone and greasy sebaceous material, and golden-yellow appearance of lower pole renal mass (black arrow)

Grossly (Figure 2), the cyst wall appeared irregular with areas of congestion. On incision, large amounts of yellow-white sebaceous and keratinaceous material were noted, along with the presence of a tooth and a bony fragment. The circumscribed, multinodular renal mass with intact capsule was incised longitudinally, revealing a golden-yellow cut surface (Figure 2).

**Figure 3:**
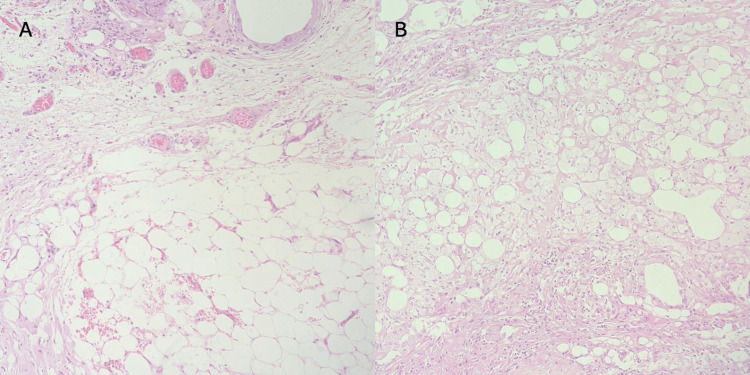
Microphotographs showing sections of an inflamed mature teratoma (A) Adipose tissue and inflammatory cells seen in the wall of the adnexal mass (H&E: 400×). (B) Seromucinous glands noted in another section (H&E: 400×) H&E: hematoxylin and eosin

Histopathological examination (Figure 3A, 3B) of the adnexal mass showed features suggestive of an inflamed and ulcerated mature cystic teratoma. The renal carcinoma was of clear cell type and confined to the kidney, with grade 1 histomorphology as classified by the freely available and widely used World Health Organization/International Society of Urological Pathology (WHO/ISUP) grading [7].

## Discussion

Mature ovarian teratomas, or dermoid cysts, comprise the largest group of ovarian germ cell tumors in women of reproductive age, although they are known to occur in premenarchal and postmenopausal groups as well [8]. Most are asymptomatic and detected on examination or routine imaging, while others present with chronic pelvic pain, an abdominal mass, or acutely with torsion. The rarer surgical complications include rupture presenting with peritonitis, and small bowel obstruction.

Bowel obstruction due to benign dermoid cysts has been reported scarcely in the past and with varying mechanisms [2-6]. Extraluminal compression by mass effect has been observed [2]. There are reports of fistulous communications between the bowel and teratoma [3], resulting from infection and rupture, or malignant transformation [9]. Rupture can cause chemical peritonitis and therefore adhesive obstruction, sometimes mimicking peritoneal carcinomatosis [4]. The case in question was that of an unruptured dermoid leading to SBO; it has been hypothesized that repetitive, incomplete torsion of the cyst leads to necrotic and degenerative wall changes [5]. Subsequent pericystic inflammation predisposes to adhesive SBO.

Due to its prevalence as an incidental finding, at an initial presentation of obstruction, the role of an ovarian dermoid may not be suspected. However, certain CECT findings aid in the diagnosis; multiple air foci and intracystic oral contrast suggest fistulization [10]; peritoneal fat implants and abscess cavities suggest rupture [4]. Surgical exploration is confirmatory in the absence of such clues. Management consists of the surgical removal of the cyst with repair of the bowel wall if required, and it is imperative to rule out the malignant transformation of the dermoid on histopathology.

Thus, a high index of suspicion is required to diagnose the etiological role of this common incidental finding in bowel pathologies. Additionally, although there have been reports of renal cell carcinoma metastasizing to the ovary, this is, to the best of our knowledge, the first documentation of a renal cell carcinoma synchronous with a benign ovarian dermoid cyst [11].

## Conclusions

This case report aims to highlight small bowel obstruction as a rare presentation of a benign ovarian dermoid cyst, with the unusual simultaneous presence of ipsilateral renal cell carcinoma. With the universal application of cross-sectional imaging in the evaluation of surgical patients, it is inevitable to come across incidental masses. However, it is simultaneously difficult to determine the causal role of such an “incidentaloma” in an acute presentation. In the present case too, it was unknown at initial presentation whether any correlation existed between the triple morbidities of small bowel obstruction, a right adnexal mass, and an ipsilateral renal mass. Therefore, incidental findings may sometimes prove to be relevant to the clinical context, and it is the knowledge of such unusual associations that can help the surgeon negotiate their way through the imaging findings of incidentalomas.
